# Ejection Behavior of Commercial Hydrogels with Potential Use for Biomedical Applications via In Situ Bioprinting

**DOI:** 10.3390/gels12050401

**Published:** 2026-05-06

**Authors:** Sirje Liukko, Katarina Dimic-Misic, Milica Marceta Kaninski, Michael Gasik

**Affiliations:** 1 Department of Chemical and Metallurgical Engineering, School of Chemical Engineering, Aalto University, 02150 Espoo, Finland; 2 Institute of General and Physical Chemistry Belgrade, 11000 Belgrade, Serbia; katarina.dimicmisic@gmail.com (K.D.-M.);

**Keywords:** apparent viscosity, soft tissues repair, ejectability, hydrogel, nanocellulose, in situ bioprinting

## Abstract

For personalized treatments, including soft tissues repair, the use of in situ bioprinting is of increased interest. Many soft tissues, such as sphincters, have poorly known mechanical properties and a complex structure, with limited options for a medical practitioner to assess where the injections should be made and how much should be injected. The rate of injection and its variation have a direct implication on pain sensation for patients, but post-injection efficacy largely depends on the ability of the hydrogel to adapt to local loads and displacements, keeping the 3D structure compliant to the surrounding tissues. Such a method is known as ‘in situ bioprinting’. There are, however, limited data regarding hydrogels’ functionalities for such applications, and many commercial hydrogels, as medical devices, are used off-label. This study aims to introduce an innovative, robust, and reliable approach for evaluating the ejection-related mechanical properties of various commercial hydrogels. The ejectability of six clinically approved hydrogels was assessed through their rheological properties, characterized by measuring apparent viscosity using a mechanical testing device in a novel setup combined with the dynamic syringe pump analysis (for a pre-set constant ejection rate). It was shown that a well-established power-law approximation offers a straightforward, less computationally intensive approach than more complex models that attempt to account for viscosity, shear rate, and wall slip. It assesses hydrogel performance within an actual system, including the syringe and nozzle, rather than just characterizing the material in isolation, thus making it particularly valuable for predicting how gels will behave under real conditions. This method can be adapted for specific clinical bioprinting applications, including sphincter repair, lipoatrophy correction, or deep dermal/transdermal targets, optimizing speed, flow rate, and applied force.

## 1. Introduction

Hydrogels are three-dimensional hydrophilic polymer networks that contain and retain aqueous media [1]. Their polymer phase can be synthetic or natural, the latter derived from polysaccharides or proteins. Natural polymers provide feasible features for hydrogels, such as flexible and soft structure, porosity, biocompatibility, and biodegradability [2,3]. Hydrogels have already illustrated their diversity, which makes them next-generation biomaterials for biomedicine, tissue engineering, novel treatment protocols, and smart drug delivery [3]. Hydrogels are a practical way for dispersing solid components that may otherwise settle quickly. Another branch of biomedical applications of hydrogels are carriers for a long-acting, sustained-release of medication, such as psoriasis treatment or various wound dressings [4,5]. Surgery applications can utilize various handheld extruder devices for biomaterials [6]. For all of these applications, mechanical control of flow is essential for successful results.

Clinically used hydrogels typically have a low particle density, and describing or modeling their rheology is challenging because their stress–strain response is influenced by their complex microstructures and differences in solids content. Although there are several clinically approved hydrogel-based products on the market, their actual mechanical behavior under physiologically relevant conditions is rarely studied in publications, despite the fact that these parameters are essential for clinical applications.

A fast-developing advancement of utilizing hydrogels is in bioprinting [7,8]. The behavior of hydrogels in bioprinting, including flow parameters, and post-printing properties, such as shape retention and shape adaptation, is critical for the process (Table 1). The most applied 3D printing technique is extrusion type, where bioinks loaded in the dispensing head are extruded by either pneumatic or mechanical forces, through a print needle, to form continuous filaments. By controlling the dispensing head’s movements, positioning, and temperature through a computer interface, 3D structure will be stacked layer by layer following the design [9]. During the bioprinting process, the critical biomechanical factors include the rheological properties of the bioinks, surface tension, regulation of the flow rate, and the mechanical forces generated during printing. In extrusion-based 3D bioprinting, a major challenge lies in identifying suitable hydrogels and establishing reproducible procedures that ensure high printability while producing constructs with excellent shape fidelity, close similarity to the designed model, and tunable mechanical properties [10]. When live cells are added to the bioink, they must maintain their viability after passing the narrow extrusion nozzle and high shear stress zone. Hydrogels composed of mixtures of synthetic and natural polymers allow for the optimization of their physicochemical and biological characteristics, thereby improving scaffold performance.

In customized treatment, when this bioprinting is being done in situ (directly into the patient), both painless injectability and shape adaptation are very important [11,12,13]. This is different from conventional injection of a hydrogel into the target site with the assumption that it will fill proper parts itself (which may or may not be true). However, challenges such as shape mismatch, unstable adhesion, and contamination arise during the transition from externally printed scaffolds to their internal transplantation, thereby limiting clinical applications. Compared to traditional 3D bioprinting, in situ 3D bioprinting offers enhanced tissue integration and surface construction capabilities, demonstrating greater clinical potential [14]. In situ bioprinting already foresees that injected material adopts a certain shape and will retain sufficient mechanical properties. In soft materials bioengineering, more challenges persist in printing and integrating fibers or solids into hydrogels to mimic biological systems and to reach necessary conditions. The goal of achieving high-resolution, multi-material 3D hydrogel structures continues to push the limits of precision and scalability in 3D fabrication. Depending on the target, different syringes and needles can be used even for the same material, and the rate must be maintained to avoid excessive pressure (pain control, failed procedure caused by overfilling, and necrosis prevention) and to achieve anastomosis.

**Table 1:** Key properties, functions, and benefits of hydrogels in 3D bioprinting applications [1,2,3,15].

Property	Function	Benefits for Bioprinting
Bioactive properties	Hydrogels can be loaded with bioactive molecules, such as growth factors, peptides, and proteins, to enhance cell differentiation, proliferation, and tissue formation	Important for mimicking the biological environment of native tissues
Biocompatibility	Implantable hydrogels to support cell growth and tissue formation	Enhances interaction with live cells
Hydrophilicity	Water content similar to natural tissues	Provides an ideal medium for maintaining cell viability during and after the printing process
Mechanical	Soft and flexible nature	Suitable for mimicking the mechanical properties of soft tissues. Provides an appropriate environment for cells to thrive and differentiate
Printability	Advanced hydrogel chemistry has resulted in materials that possess desirable flow and curing characteristics	Suitable for being extruded through a bioprinter’s nozzle. Can be engineered to have shear-thinning properties, which help maintain the integrity of printed structures
Tunability	Mechanical and biochemical tailoring. Modifying factors such as crosslinking density, polymer composition, and degradation rates	Creating tailored hydrogels with properties suited to different types of tissue
Versatility	Incorporate a wide range of materials: natural polymers like collagen, alginate, and hyaluronic acid, or synthetic polymers, or solids or nanocomposites like nanocellulose	Vast choices of customization based on the specific tissue or structure type targeted for bioprinting

Incorporation of nanoparticles can enhance hydrogel stability and introduce additional biological functionalities. For example, clay-containing hybrid hydrogel (1.4–1.7%) inks were reported [16] with improved rheological properties. These enforced di-block copolymer hydrogels, after extrusion and 3D printing, exhibited significant improvement in shape fidelity and enhanced shear-thinning character, in combination with rapid viscosity recovery and structure recovery, characteristics that are highly beneficial for ejection-based 3D printing production. Different types of materials may require different parameters [16]; and, thus, a universally predictive rheology parameter for good printability has not been found yet. Viscosity of hydrogels used for the purpose is one of the important features of the process. Hydrogels can be categorized as chemically crosslinked or physically crosslinked, while composite hydrogels are formed from two dissimilar materials [2,17]. Selection criteria for prominent hydrogels for this study prioritized the commercially available hydrogels with variations in their expected viscosity and versatility of their solids, such as fibrous, mineral, or polymer solid fraction. For Orthovisc (viscosupplementation) and Prolaryn (vocal folds’ correction), their choice was based on either the lowest expected viscosity (Orthovisc as a typical hyaluronate gel) or the presence of cellulose (Prolaryn—to compare with new GrowDex, which is based on nanofibrillar cellulose (NFC)).

In an ideal Newtonian fluid, viscosity remains constant and is independent of time, but hydrogels fall within the non-Newtonian category. These materials exhibit time-dependent changes in viscosity under constant stress. When viscosity decreases with time, the fluid is described as shear-thinning (thixotropic), becoming progressively more fluid-like; blood represents a classic example of this behavior [18,19]. When viscosity increases with time under constant force, the fluid is characterized as rheopectic, where flow resistance gradually rises. Classical examples of this behavior include synovial fluid and hyaluronan-based gels. In classical rheological models, viscoelastic behavior can be considered a mixture of elasticity and viscosity using the spring–dashpot approach, which provides lumped elements as rational representations of rheological phenomena but requires fitting parameters [20]. It was suggested [21,22] that viscoelastic materials show a rheological response intermediate between elasticity and viscosity. The viscous behavior of the hydrogels dissipates energy over time, resulting in time-dependent mechanical phenomena, such as stress relaxation or creep. For real-world applications, fractional calculus-based [23,24,25,26,27] approaches have been demonstrated suitable for viscoelastic systems, such as polymeric melts, complex liquids, and memory foams. This method was deployed in injectability test protocols [28]. Several authors [29,30] have concluded that the rheology of cellulose nanofiber hydrogels is usually constrained to empirical engineering equations, but it was also shown that fractional rheology successfully described the viscoelastic phenomena in colloidal hydrogels containing TEMPO-oxidized cellulose nanofibers [30,31,32]. The concentration of a hydrogel has a direct influence on its rheological properties, while factors such as the pH of the liquid phase, zeta potential, and particle size distribution further modulate the rheological behavior of colloidal systems [33]. Owing to the complexity and variability of these parameters, no universal model currently exists to describe the rheological behavior of commercial hydrogels. It is also noteworthy that many experimental data are reporting oscillatory rheology experiments, which are much less relevant to the ejection (extrusion) type of the process, where there is neither frequency nor reciprocal strain.

Ejection performance has been evaluated for different types of commercial hydrogels that researchers aim to use in applications for soft tissues to assess their suitability for 3D bioprinting, especially 3D in situ bioprinting, when the gel is directly deposited into the patient’s tissues for repair. For this purpose, the apparent viscosity of hydrogels after ejection was measured using testing methods traditionally applied for the assessment of mechanical properties of soft materials. The novelty of this work lies in the experimental approach, where rheological behavior and structure recovery after ejection are interpreted as pseudo-mechanical characteristics, providing new insight into hydrogel performance during 3D printing.

## 2. Results and Discussion

The DMA sample probe displacement length (µm) was observed with respect to the duration of the experiment (experiment time) for applied force (N), and results were analyzed using Proteus 6.1 software (Netzsch Gerätebau GmbH, Selb, Germany). In this DMA the probe was also simultaneously used as a sensor with magnetic bearings, leading to a very high digital resolution of ±0.5 nm. Due to accumulated history, direct application of the method [28] is not feasible, as the amount of material was limited. Force segments with no or very little flow were ignored due to high potential error when displacements are less than 50–100 µm, and only those with a clear visible flow and linearity were processed. One of the most straightforward methods is in using power-law approximation (Ostwald–de Waele relationship). This equation postulates that the shear stress required to shear a fluid at a given rate can be described as

(1) $$\tau =K{\left(\frac{du}{dy}\right)}^{n},$$

where τ is the shear stress, *du*/*dy* is the shear rate, *K* is flow consistency index (factor), and *n* is flow behavior index [34]. Hydrogels are well known to display power-law viscosity and shear thinning behavior in the linear viscoelastic regime [20,35]. Power-law behavior has also been observed in the nonlinear viscoelastic regime, where the hydrogels with fibrous filaments in their matrix may show, at high shear rates, stiffening and energy dissipation under higher stress [36], so Equation (1) is usually a good practical approximation for studying different materials.

The Weissenberg–Rabinowitsch–Mooney (WRM) correction [34,35,37] adjusts the non-uniform velocity profile in the syringe flow channel by correcting differences in viscosity distribution due to uneven shear rate distribution, termed ‘wall slip’, near the wall, resulting in a much higher shear rate at the wall. For non-Newtonian fluids, this correction due to wall slip is (Equation (2))

(2) $$\dot{\gamma }=\left\{\frac{\left[3n+1\right]}{4n}\right\}\;{\dot{\gamma }}_{D},\;{\dot{\gamma }}_{D}=\frac{8V}{D}.$$

where *n* = *d*[*log t_w_*]/*d*[*log* (*8V*/*D*)] is the slope of the measured *log*(*t_w_*)—*log* (*8V*/*D*) curve; *t_w_* is the shear wall stress; *V* is the flow velocity; and *D* is the diameter of the outlet orifice. With WRM correction, the logarithm of the apparent shear rate is plotted against the logarithm of the wall shear stress, and the slope of this relationship corresponds to the fluid’s power-law index (*n*). However, in the present work, it was found that use of the WRM correction for capillary flow, as is the case of the syringe, was not always applicable, since empirical data had evident flow segments where the slope would be *n* < −1/3, due to kinetics and time-depending creeping phenomena in the system, and therefore the log-plot was not possible.

Another method described by By White and Harvey [38] related pressure drop and flow through a circular tube as

(3) $$Q=\frac{\pi r^{3}}{\frac{1}{n}+3}{\left(\frac{\Delta pr}{2LK}\right)}^{1/n},$$

where *Q* is the volumetric flow rate, *r* is the radius of the capillary, *L* is the length of the capillary, and Δ*p* is the pressure difference applied. Reordering Equation (3) gives logarithmic form (Equation (4)),

(4) $$\log\left(\frac{\Delta pr}{L}\right)=n\log\left(\frac{Q}{r^{3}}\right)+\log\left(2K\;{\left[\left(\frac{1}{n}+3\right)\;\;\frac{1}{\pi }\right]}^{n}\right),$$

from which slope *n* and the intercept can be determined. Here, *K* has a similar meaning as for Equation (1), but it is calculated in a different way and, in general, may have different values compared to the above. Equation (4) above resulted in curves from which it was easier to obtain *n*-values than Equation (3) but showed that linearity in the logarithmic coordinates was not always possible to get (similar challenges when *n* < −1/3), and no consistent data were obtained for all segments of interest.

For simple power-law approximation, a very good linearity was obtained for displacement slope (µm/min) vs. time at fixed applied forces (Table 2). Converting these values into stress and strain rate allowed for the determination of *n* and *K* values. Factor *K* combines the viscosity of the material (*η*), so by rearranging this (Equation (1)), the viscosity of the materials in this specific syringe system can be calculated as

(5) $$\eta =K{\dot{\gamma }}^{\left(n-1\right)},$$

(6) $$\log(\eta )=\log\left(K\right)+(n-1)\log(\dot{\gamma }),$$

where *K* is the flow consistency index, *n* is power-law exponent, and $\dot{\gamma }$ is the shear rate in the definition specified by Equation (1). In the syringe pump experiments, the flow rate was held constant, making it impossible to directly plot viscosity versus shear rate. However, since the pressure was continuously recorded, the apparent viscosity could be calculated using a method analogous to (1) and (2). The resulting viscosity values were then plotted against time, providing a direct correlation with the measured pressure throughout the experiment.

It is important to recognize that hydrogels are non-Newtonian fluids, and the test setup introduces multiple additional factors, including system friction, non-uniform flow, transition of the gel structure, and the change in geometry from wider diameter to narrow needle channel. Many of these factors are difficult to quantify, but for practical application, the overall system response is the most relevant measure. To address the complexity and versatility of these materials, a simple power-law-based empirical method was deployed as a practical approach for characterizing hydrogel properties. As explained above, the complex hydrogels display power-law viscoelasticity in the linear viscoelastic regime. Still, the same has been observed in the nonlinear viscoelastic regime, where hydrogels with fibrous filaments may show stiffening and energy dissipation under higher stress. In other words, these commercial hydrogel samples can be described as nonlinear hydrogels with asymmetrical mechanisms that, under force or pressure, exhibit mechanical behavior in which their response to stress or strain is not directly proportional. This means that they can show nonlinear resistance to deformation changes as we apply force under testing, thus mimicking the force on hydrogels during the 3D printing.

**Figure 1:**
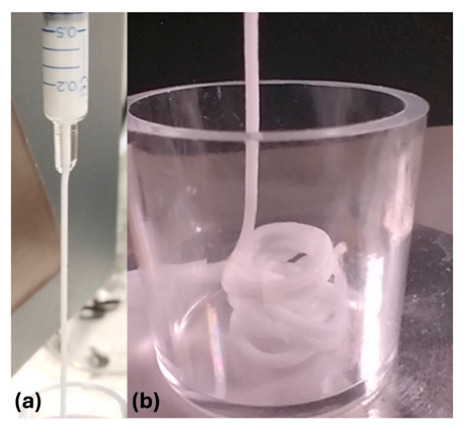
Photograph of ejected Coaptite^R^ hydrogel from syringe: (**a**) Needleless syringe is placed as free-standing within DMA holder (Figure 1), under the influence of a force applied with a stepwise creep mode, and (**b**) ejected hydrogel that simulates 3D printing process.

**Figure 2:**
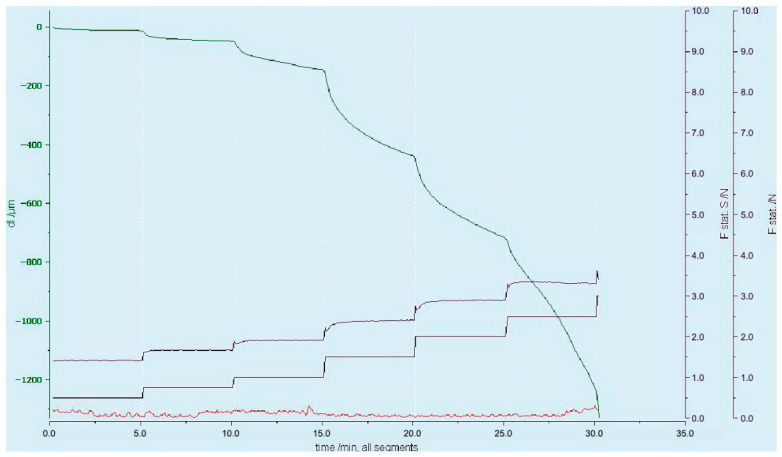
A screenshot of the stepwise creep test of a syringe with a gel showing displacement of the probe (μm), static force on sample (N), and programmed applied force (the differences between them are due to subtraction of the sample’s holder stiffness correction). The line colours correspond to the variable axes shown in the same colour.

Applying mechanical force on hydrogels of non-Newtonian types under well-controlled experimental environment and mechanical test setup is the methodology of our choice. Through calculations, the empirical data can reveal the actual mechanical differences between these complex hydrogels and whether they stiffen or soften under stress. For bioprinting applications, it is vital to know the force ranges and relative times needed for hydrogels to flow from a syringe. Figure 1 displays the photograph taken during the testing of hydrogel in a syringe placed free-standing into the DMA holder and applying creep mode. The test force was increased in a stepwise manner, resulting in segmented flow data (Figure 2).

The screenshot of the stepwise creep test data is presented in Figure 2. This was obtained by positioning the free-standing syringe into a DMA holder and ejecting pressure on top of the piston applied in a creep mode with a stepwise increase in force from 0.1 to 11 N.

Table 2 summarizes the data for all analyzed samples across the segments with constant applied force where stable and clearly observable flow was achieved. The corresponding flow behavior, expressed as displacement per unit time versus applied force, is illustrated in Figure 2.

**Figure 3:**
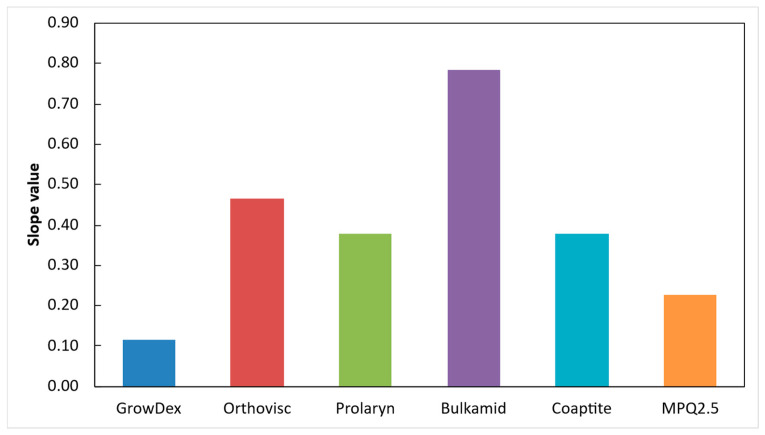
All tested hydrogels and their power-law-fit-based empirical slope values (Table 2).

Figure 3 shows that the GrowDex hydrogel has the lowest shear-thinning coefficient (*n*) obtained from the power-law fit, whereas the Bulkamid gel exhibits the highest *n* value. This indicates that GrowDex displays the most pronounced shear-thinning behavior, characterized by a substantial reduction in viscosity under applied shear. In contrast, Bulkamid demonstrates the weakest shear-thinning effect, with viscosity remaining comparatively less sensitive to increasing shear stress. In Figure 3, shear-thinning coefficients for all hydrogels are shown according to (1). When the *n* coefficient is high, the material reacts stronger to the increase in applied stress.

**Figure 4:**
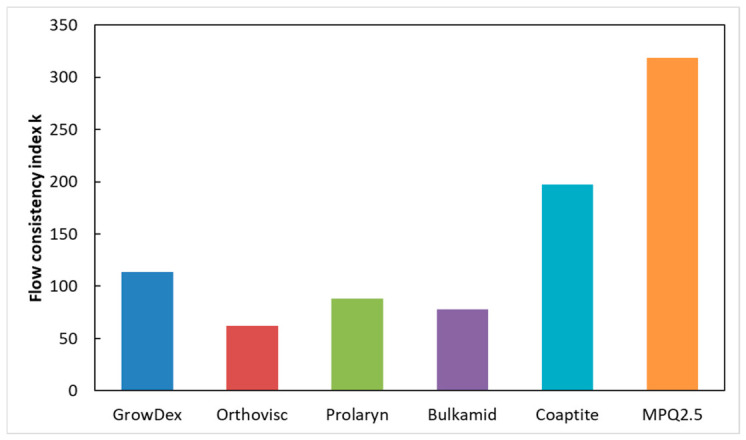
All tested hydrogels and their power-law-fit-based flow consistency index (Table 3).

Figure 4 presents the flow index (*K*) according to the power-law model and the corresponding shear-thinning coefficient (*n*). Higher *K* values indicate slower flow behavior and higher consistency of gels, and *K* values are highest for gel MPQ2.5 and smallest for Orthovisc. One must, however, keep in mind that this analysis is based on an approximate model with inherent simplifications for describing non-Newtonian fluids. Consequently, the viscosity values obtained from (5) and (6) in Table 2 should be interpreted as relative comparisons between materials under identical conditions, rather than absolute viscosity measures. For this reason, the term ‘apparent viscosity’ is used throughout this discussion.

**Table 3:** Summary of the tested hydrogels, product names, and their composition, with listed clinical or biomedical uses.

Product Name	Composition	Intended Clinical Use	Visual Appearance
GrowDex^R^	3.0% nanofibrillar cellulose, sterile water	3D cell culture matrix, drug release	hand cream
Orthovisc^R^	1.5% purified Na-hyaluronate, sterile saline	Osteoarthritis joints pain (viscosupplementation)	hand wash
Prolaryn Gel^R^	Na-carboxymethylcellulose, glycerin, phosphate buffer, sterile water	Vocal folds’ insufficiency	hand sanitizer
Bulkamid^R^	2.5% polyacrylamide, sterile water	Bulking agents for stress urinary incontinence (SUI)	jellyfish
Coaptite^R^	Hydroxylapatite (75–125 µm), glycerin, Na-carboxymethylcellulose, sterile water		stiff toothpaste
Macroplastique^R^ MPQ2.5	Medical-grade polydimethylsiloxane, sterile water		sugared honey

**Figure 5:**
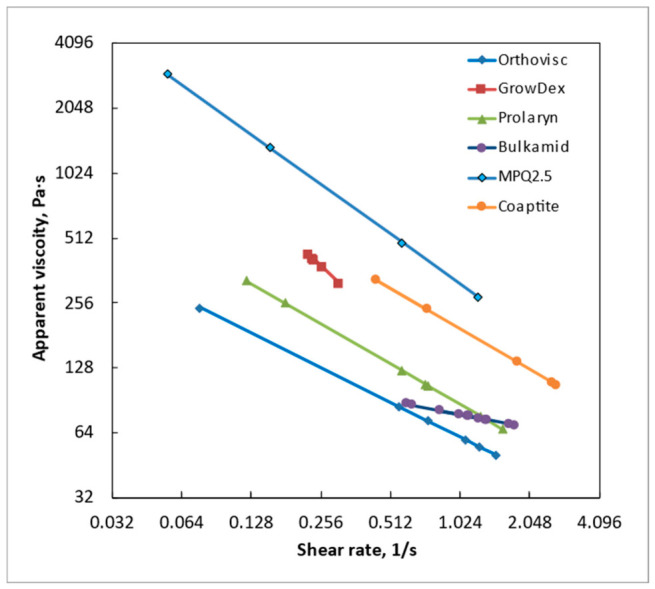
All tested hydrogels and their apparent viscosity as function of shear rate.

Figure 5 shows the decrease in apparent viscosity (*η*) for all gels during ejection from the syringe. This reduction in η reflects how each material responds to applied shear under conditions that simulate practical injection or dispensing. As indicated in Table 1, the gels display distinctly different slopes in the decline of apparent viscosity, highlighting notable variations in their ejectability. A steeper decrease in η suggests that the material becomes significantly less viscous under shear, enabling smoother and easier flow through the syringe needle. Conversely, gels with a more gradual decline in viscosity maintain greater resistance to flow, indicating more challenging ejection behavior and potentially requiring higher injection force.

**Figure 6:**
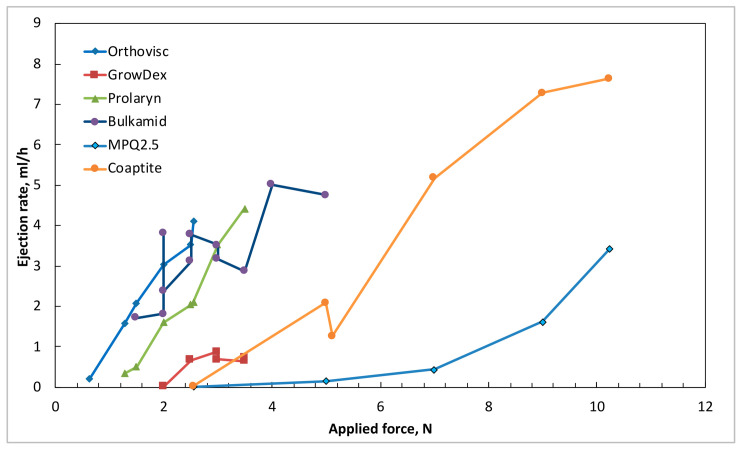
All tested hydrogels and observed ejection rate (mL/h) as function of applied force.

The ejection rate, a parameter presented in Figure 6, is for gels extruded (ejected) through a 23G syringe needle, representing the speed and consistency with which a material flows under applied pressure, serving as an indicator of its practical usability. This rate is directly influenced by the rheological properties of a material—particularly shear-thinning behavior and apparent viscosity—and determines how easily the gel can be extruded without interruption. A higher ejection rate indicates that the material experiences a substantial decrease in viscosity under shear, allowing it to flow smoothly with minimal force, while a lower ejection rate suggests greater internal resistance and the need for higher pressure to achieve extrusion. Understanding and optimizing the ejection rate is especially critical in 3D printing and bioprinting, where precise, continuous, and controlled material deposition is essential for producing accurate structures.

Materials with well-regulated ejection behavior enable uniform formation, improved printability, and high shape fidelity, preventing issues such as clogging, spreading, or irregular strand thickness. Moreover, consistent ejection ensures reproducibility across prints and compatibility with printer hardware, while in bioprinting applications, it helps protect embedded cells from excessive shear stress. The ejection rate is a critical rheological parameter that strongly determines the quality, precision, and overall success of extrusion-based 3D printing processes. It governs how consistently a material can be deposited, directly influencing filament stability, structural fidelity, and printing reliability.

The results of this study demonstrate that Bulkamid hydrogel behaves markedly differently from all other tested materials. It shows the steepest slope in response to applied stress and exhibits significant instability in its flow behavior (Figure 3, Figure 4 and Figure 5). Under identical applied forces, the shear rate of Bulkamid fluctuates by a factor of 2–3, and at higher pressures, it even tends to decrease, indicating an inconsistent shear-thinning response. Such irregular flow behavior implies that Bulkamid does not shear-thin as predictably as the other hydrogels, which can compromise controlled and uniform extrusion. As supplementary information, the authors provide a video of the Bulkamid ejection (Supplementary Information Video S1) as a visualization of the findings stated of this research. These deviations are likely linked to Bulkamid’s distinct composition, primarily polyacrylamide (Table 1), which sets it apart from the other hydrogels analyzed. Although its viscosities (Table 2; Figure 5) fall within a similar range to those of Orthovisc and Prolaryn, viscosity alone cannot fully account for its unusual behavior. Instead, the interaction between its polymer network architecture and its non-Newtonian flow characteristics likely contributes to the observed instability. This highlights the importance of assessing multiple rheological descriptors—not only viscosity—when determining material suitability for extrusion-based manufacturing. The exact causes of Bulkamid’s limited shear-thinning and unstable flow remain unclear; however, reduced shear-thinning capacity may significantly hinder its ejection and overall usability in bioengineering or filler applications, as uneven flow prevents the formation of continuous, well-defined strands. In contrast, Macroplastique (MPQ 2.5) exhibits extremely high viscosity, making it difficult to inject and unsuitable for 3D bioprinting. Coaptite shows more favorable performance, offering reasonable ejection rates together with good post-extrusion shape retention (Figure 6).

Orthovisc and Prolaryn exhibit similar rheological profiles, and their viscoelastic properties appear well-balanced for applications requiring tissue correction. GrowDex gel presents a particularly useful combination of characteristics: it has a moderate slope and low shear-thinning coefficient, resulting in a relatively stable apparent viscosity over a narrow operational window (Figure 5 and Figure 6). As supplementary information, the authors provide video of the GrowDex ejection (Supplementary Information Video S3), showing evenly filling flow. The reasons behind th stability of GrowDex cannot be attributed solely to the nanofibrillar cellulose structure [29,30,31,32]; nevertheless, it may explain the consistently favorable reports on the use of GrowDex-based materials as 3D bioprinting inks [39]. These findings reinforce the need for comprehensive rheological evaluation when selecting materials for extrusion-based 3D printing, as subtle differences in composition and flow behavior can significantly impact printability, structural fidelity, and application-specific performance.

**Figure 7:**
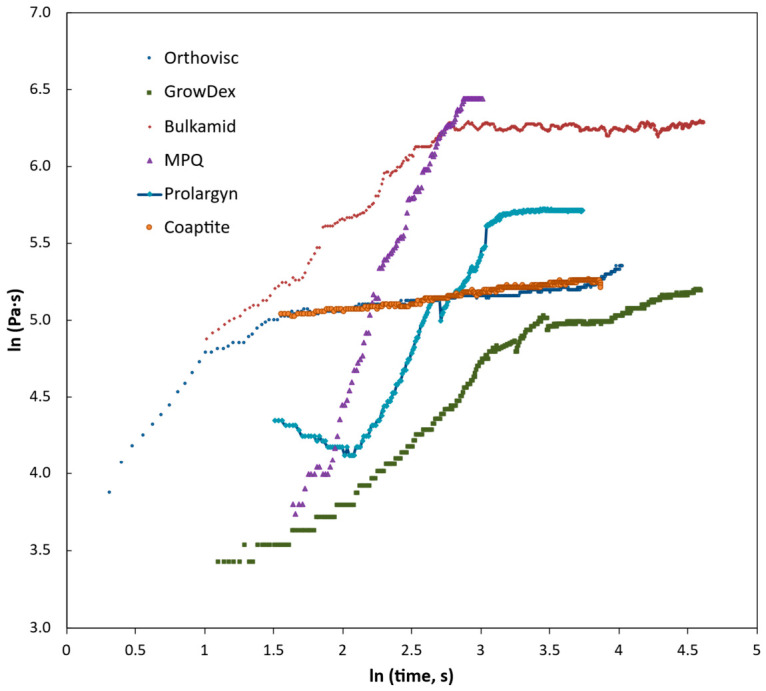
Logarithm of apparent viscosity vs. time of ejection under 0.36 mL/min flow rate through 23 G needle.

In the syringe pump experiment, the relationship between the logarithm of viscosity and log time is presented in Figure 7. For nearly all hydrogels, two distinct regions can be observed: an initial ramp phase, where viscosity changes rapidly as flow begins, followed by a plateau region, which corresponds to a steady-flow state. However, the MPQ gel required pressures higher than those that could be generated by the sensor’s 30 N force limit. As a result, the ‘plateau’ seen in the MPQ curve does not represent actual steady flow but rather a limit imposed by the instrument. The kinetics of the ejection as force vs. time for the rate of 0.36 mL/min can be seen from the ‘free ejection’ Supplementary Information Video S4. Coaptite exhibited a relatively gentle ramp slope, producing a plateau like that of Orthovisc gel, though slightly weaker. Prolaryn gel, on the other hand, showed unstable behavior characterized by sudden jumps in pressure and apparent viscosity. These fluctuations were not caused by testing artifacts such as sensor misalignment or trapped air bubbles, and the source of this instability remains unclear. Notably, this behavior differed from what was observed during the constant-pressure creep test, suggesting that Prolaryn gel responds differently depending on the loading conditions. Under the same experimental conditions, GrowDex showed the lowest viscosity among all samples once steady flow was reached. This may indicate that GrowDex possesses strong shear-thinning properties, making it more suitable for processes that rely on a consistent shear rate, while being less favorable for constant-pressure (creep) conditions [39,40].

**Figure 8:**
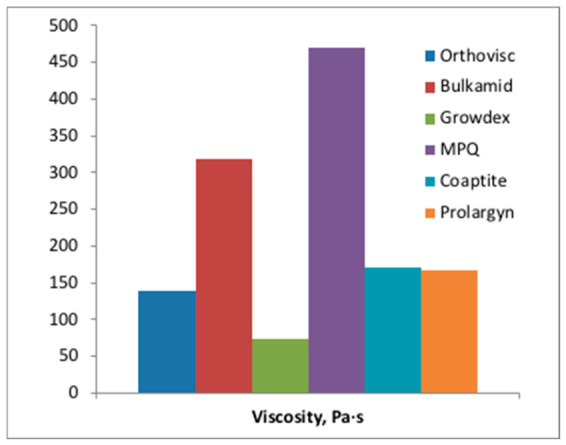
Estimated viscosities of the gels at steady flow conditions (0.36 mL/min, 23 G needle).

Based on the steady-flow (plateau) regions, apparent viscosities were estimated (Figure 8). MPQ gel and Bulkamid gel displayed the highest viscosities in this regime, which could pose challenges for in situ bioprinting applications. Such highly viscous gels require substantially greater pressure to maintain flow, and when cells are incorporated, the heightened shear stresses inside the needle may compromise cell viability. To observe post-ejection behavior, gels were also ejected into an empty syringe with recording (Supplementary Information Videos S1–S3). Supplementary Information Video S1 shows that Bulkamid has a tendency to form clusters after entering free space off the needle, whereas Orthovisc (having least viscosity) eventually tends toward uniform filling (Supplementary Information Video S2). Growdex first forms a ‘tree’ of clusters but readily fills space completely after that (Supplementary Information Video S3).

Overall, these findings emphasize that when working with hydrogels in extrusion-based systems—including 3D bioprinting—it is essential to consider how the material behaves in the actual printing hardware, not only in controlled rheometer conditions. Factors such as friction within the syringe, mechanical creep, and interactions between the gel and nozzle can significantly influence flow performance. These effects are not directly captured by the conventional rheometry, highlighting the importance of evaluating materials under application-relevant conditions. Estimated apparent viscosities calculated under the applied conditions—using the flow index (*K*) and the shear-thinning index (*n*) from the power-law model, presented in Figure 8, provide critical insight into how easily each gel can be extruded through a syringe or nozzle. These parameters determine how the material behaves under shear: gels with lower *K* values and stronger shear-thinning behavior (lower *n*) exhibit reduced viscosity during flow, improving ejectability and enabling smoother, more controlled extrusion. In the context of bioprinting, this is essential for achieving consistent filament formation, maintaining structural precision, minimizing pressure requirements, and protecting embedded cells from excessive shear stress. High viscosity is generally not suitable for printing of gels because it requires greater force to extrude through a syringe or nozzle (for hand-driven process, forces <22 N are recommended), making continuous flow difficult and prone to clogging. It also reduces the material’s ability to shear-thin under stress, leading to uneven extrusion and inconsistent filament formation, thus compromising shape fidelity and structural accuracy. In bioprinting applications, high viscosity can increase local shear stress during extrusion, potentially damaging or killing embedded cells. Additionally, very viscous gels can strain printer components, further limiting their practical use in extrusion-based bioprinting.

## 3. Conclusions

In this study, a novel and practical method for evaluating the apparent viscosity and ejectability of commercial hydrogels was developed and quantified, providing an essential tool for extrusion-based applications such as 3D (bio)printing. By using a well-established power-law approximation, this method offers a more straightforward, less computationally intensive approach than more complex models that attempt to account for viscosity, shear rate, and wall slip—models that can fail under log-transformations due to data scatter or local negative values. The significance of this method lies in its ability to assess hydrogel performance within an actual bioprinting system, including the syringe and nozzle, rather than just characterizing the material in isolation. This makes it particularly valuable for predicting how gels will behave under real extrusion conditions, which is crucial for ensuring reliable filament formation, consistent flow, and successful in situ bioprinting.

Testing six commercial hydrogels demonstrated how this approach can identify key parameters necessary for controlled flow, helping establish protocols that replicate printing conditions using mechanical testers such as DMA. While this study was limited to a single syringe type and diameter, the method can be adapted for specific clinical bioprinting applications. Traditional rheological measurements obtained under oscillatory conditions may not reflect real extrusion behavior. In contrast, this apparent viscosity evaluation provides a more accurate, application-relevant assessment that guides both material selection and process optimization for practical bioprinting scenarios.

## 4. Materials and Methods

### 4.1. Methods of Measurements

The Dynamic Mechanical Analyzer DMA242E ‘Artemis’ (Netzsch Gerätebau GmbH, Selb, Germany) has a built-in moving-magnet drive for precise force measurements from 0.5 mN to 8 N to characterize the dynamic mechanical behavior of various types of materials with high precision. The details of this method are described in detail elsewhere [28]. Briefly, clean syringes of 1 mL capacity (internal diameter 1/4″) without needles (outlet inner diameter, 1 mm; channel length, 10 mm) were filled with ~0.5 mL of the gels. The syringe geometry remained constant through the tests. Needleless setup was chosen because different gels’ packages are supplied with very different gauges (from G10 to G27) and length, and some of high-viscosity ones were not possible to eject (with force > 30 N) when the needle was used.

**Figure 9:**
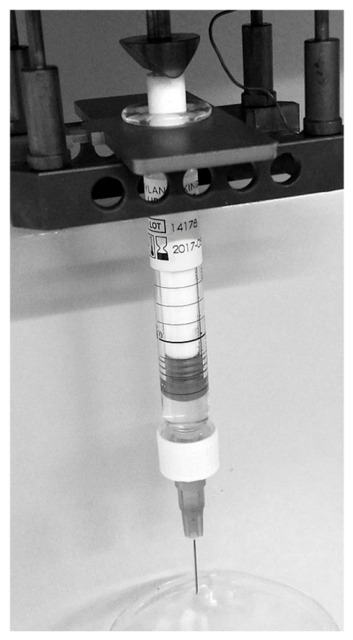
Schematic of the ejection testing-sample holder in DMA. Similar syringes were tested for all hydrogels without a needle.

The syringes were positioned to be free-standing in a DMA holder, as illustrated in Figure 9, and ejecting pressure on top of the piston was applied in a creep mode with a stepwise increase in force from 0.1 to 11 N (depending on the gel type; Figure 1). Due to the system geometry design, traditionally used Bagley, Couette–Hagenbach, and Lupton slip corrections were not applicable. The creep test usually used for mechanical testing of soft samples was used in several segments to observe initiation of the extrusion of sample through the syringe as a start of the gel ejection into free air. In total, 5 to 12 tests were carried out for each gel, and several segments were programmed into stepwise force increase (always from the smallest to the largest force).

The second part of the study was made with a homemade syringe pump with 1.5 mL COC syringe and 23 G × 1″ needle, when ejection was made under constant feed rate 0.36 mL/min. Syringe pump was equipped with a pressure sensor (Tekscan Inc., Boston, MA, USA), which was preliminarily calibrated with reference pressures up to a 30 N load. The pressure signal was translated via A/D converter into measurement software (ELF Tekscan 4.3.3.0) and recorded in separate files, resulting in raw data as CSV files and dynamic videos (FLF format), and the latter were converted into a standard video format. Identical syringes were used for all hydrogels, and experiments were made in triplicate; no significant differences were found, but some experiments were repeated due to misaligned contact between syringe pump and the sensor. All tests were done in a climate-controlled clean room, ISO Class 5 (USP-compliant), at a temperature of 25 ± 1 °C and relative humidity (H) of 25 ± 5%.

### 4.2. Analyzed Hydrogels

Commercially available hydrogels that were used for testing are listed in Table 3 and were assessed as received in their manufactured compositions. It is notable that the IFU that accompanied the packages did not have full, exact quantitative data on the hydrogels’ compositions. Not all the gels were indicated for sphincter repair, but there are clinical data showing that such use has been tested, although to a limited extent. For Orthovisc (viscosupplementation) and Prolaryn (vocal folds correction), their choice was based on either lowest expected viscosity (Orthovisc as a typical hyaluronate gel) or presence of cellulose (Prolaryn—to compare with new GrowDex, which is based on nanofibrillar cellulose (NFC)).

## Figures and Tables

**Table 2:** Summary of experimental data for creep.

Material	Applied Force N	Equivalent Shear Rate, 1/s	Power-Law Fit Slope, *n*	Flow Consistency Index, *K*	Apparent Viscosity (η), Pa·s
GrowDex	2.5	0.238	0.115	113.64	405
	3	0.309			321
	3	0.241			401
	3.5	0.231			416
	3.5	0.262			372
Orthovisc	0.64	0.077	0.466	62.07	244
	1.28	0.558			85
	1.5	0.740			73
	2	1.073			60
	2.5	1.248			55
	2.56	1.454			51
Prolaryn	1.28	0.122	0.378	88.17	326
	1.5	0.180			256
	2	0.571			125
	2.5	0.724			108
	2.56	0.748			106
	3	1.251			77
	3.5	1.563			67
Bulkamid	1.5	0.609	0.785	78.11	87
	2	0.642			86
	2	1.346			73
	2	0.842			81
	2.5	1.104			76
	2.5	1.335			73
	3	1.244			75
	3	1.125			76
	3.5	1.016			78
	4	1.776			69
	5	1.682			70
	6	0.767			83
Coaptite	5	0.740	0.379	197.46	238
	5.12	0.448			325
	7	1.832			136
	9	2.574			110
	10.24	2.701			107
Macroplastique MPQ2.5	5	0.056	0.228	318.55	2965
	7	0.154			1351
	9	0.574			489
	10.24	1.215			274

## Data Availability

Raw data are available upon request from the corresponding author.

## Supplementary Materials

The following supporting information can be downloaded at https://www.mdpi.com/article/10.3390/gels12050401/s1, Video S1: Ejection of Bulkamid. Video S2: Ejection of Orthovisc; Video S3: Ejection of Growdex; Video S4: Kinetics of the force increase with time for different gels.
